# The Benefits of Intergenerational Wisdom-Sharing: A Randomized Controlled Study

**DOI:** 10.3390/ijerph19074010

**Published:** 2022-03-28

**Authors:** Karl Pillemer, Julia Nolte, Leslie Schultz, Harry Yau, Charles R. Henderson, Marie Tillema Cope, Barbara Baschiera

**Affiliations:** 1 College of Human Ecology, Cornell University, Ithaca, NY 14850, USA; jn472@cornell.edu (J.N.); ls30@cornell.edu (L.S.); yauwanhung@gmail.com (H.Y.); crh2@cornell.edu (C.R.H.J.); mtc233@cornell.edu (M.T.C.); 2 Dipartimento di Filosofia e Beni Culturali, Università Ca’ Foscari, Dorsoduro 3246, 30123 Venice, Italy; barbara-baschiera@unive.it

**Keywords:** intergenerational programs, wisdom, youth development

## Abstract

Adolescents’ opportunities to benefit from the life wisdom of older persons are very limited. To address this issue, we designed and tested the Building a Community Legacy Together (BCLT) program based on research on the benefits of older people’s wisdom for youth development. In the intervention, the youth participants were trained prior to conducting interviews with older persons regarding their advice for living. The youth participants analyzed the information obtained and presented a summary report to the community. The participants were 93 middle and high school youth who were randomly assigned to the treatment condition with the BCLT program (*n* = 47) or to the control condition (*n* = 46). The outcome measures included sense of purpose, self-esteem, attitudes toward older people, confidence interacting with older people, and interest in working with older people. Quantitative and qualitative data were also collected regarding the subjective assessments of the program’s success. We found significant positive effects for the BCLT participants regarding their sense of purpose in life, attitudes toward older people, comfort interacting with older people, and interest in working with older people. The subjective assessments of the participants were overwhelmingly positive. The findings indicate that BCLT had positive effects for the youth participants and support the further development and testing of wisdom-sharing intergenerational programs.

## 1. Introduction

In 2015, adults aged 65 and older made up only 9% of the population worldwide (617.1 million) [1]. By 2050, they are estimated to represent 17% of the global population (1.6 billion), more than doubling in the span of 35 years. Paradoxically, as the older population is growing, societies are confronted by the lack of meaningful connections between younger and older people [2]. The shortage of opportunities for interaction increases the likelihood that young people will develop negative attitudes and stereotypes toward older adults based on prejudices and incorrect assumptions [3].

The lack of contact also makes it impossible for young people to learn from older people’s accumulated life wisdom. Sharing wisdom has positive effects on youthyounger persons, including assistance with the development of life skills, exposure to new relationships and opportunities, and help with overcoming obstacles and barriers [4,5,6]. To promote the optimal youth development, it is important not only to increase the frequency of contact between youth and older adults, but also to facilitate the transfer of the practical knowledge and advice [7].

Intergenerational programs (IGPs), which promote activities that bring generations together for mutually rewarding purposes, represent a potential answer to these challenges [8]. To date, however, with very few exceptions (cf. [9]), wisdom-sharing has not been studied as a mechanism for program effects. Our aim was to contribute to the knowledge in this area through an evaluation of an intergenerational wisdom-sharing program, Building a Community Legacy Together (BCLT).

One goal of this effort was to address methodological weaknesses in the IGP research [8]. First, we moved beyond anecdotal or pre–post designs by using a randomized controlled design. Second, we focused on a theoretically grounded mechanism for hypothesized effects on participants: wisdom-sharing. Third, we included outcomes that move beyond reduction of negative stereotypes and attitudes toward aging and older people, such as self-esteem and sense of purpose. Thus, although the study has a number of limitations (described below), it attempts to model the next stage of the IGP research called for by scholars in the field [8].

### 1.1. Background of the Study

Most IGPs attempt to enhance young people’s perceptions of older adults. Studies show that IGPs can increase children’s knowledge about the aging process and living as an older adult, help them become more accepting of older people, and reduce age-related stereotypes [10,11]. IGPs can reduce age-related intergroup anxiety and improve perceptions of older adults as potential friends or as easy to get along with [12]. Moreover, IGPs can enhance the likelihood of young people paying attention to and speaking with older adults [13].

These changes in young people’s attitudes are not necessarily limited to the specific older individuals with whom they interact, but are also generalizable to the older demographic at large. In a recent systematic review, IGPs were found to reduce ageism overall among young people [14]. Furthermore, engaging with older adults can increase the likelihood that youth will turn to older adults for help [15] and display a more prosocial behavior towards other people [16]. For example, youth who demonstrate improved intergenerational attitudes post-intervention may be more likely to engage in volunteering later on [17].

Other IGPs pursue changes in youth participants’ academic self-perceptions and lifestyle choices. For instance, IGPs that involve older adult tutors or mentors can enhance young people’s beliefs that they will succeed academically [18]. Further, working with an older adult can lead children to hold more positive attitudes towards school, the school climate, and the future [18,19].

Substantially less research attention has been paid to the possibility that IGP participation produces positive physical or mental health outcomes for children and adolescents, with reviews finding few investigations that measure health effects [20]. Although research is limited, studies have reported that children involved in intergenerational exchanges report better health outcomes [21] and improved psychological well-being [19] compared to children who are not. In general, IGPs may facilitate emotion regulation [16], with adolescents reporting improved positive emotions and better ego integrity post-intervention [22]. The limited literature suggests that interaction with older adults may lead to health benefits [23].

The present study advances prior research by focusing on a specific mechanism for hypothesized positive effects of an IGP for youth: the opportunity to receive advice and life wisdom directly from older people. From the earliest stages of development of IGPs, a fundamental assumption supported by empirical evidence is that older people benefit from opportunities to express generativity—making the world a better place for further generations [24]. Almost no research, however, has addressed the effects of the receipt of elder wisdom by youth participants. To fill this gap, we evaluated the effectiveness of an intergenerational wisdom-sharing intervention (BCLT) among adolescents.

### 1.2. Wisdom-Sharing as a Mechanism for Intervention

Several bodies of research suggest that wisdom-sharing may be a potent mechanism for beneficial effects in young people. The research on wisdom has flourished over the past decades, with multiple theoretical frameworks and an expanding literature on experimental psychology. This literature has shed light on determinants of wisdom, as well as its consequences for such areas as clinical judgement, occupational success, psychological well-being, and physical health [6].

Although wisdom is closely associated in the public mind with the later years, an evolving line of research has examined how wisdom may develop in adolescence and emerging adulthood. The research supports the growth potential of wisdom-sharing in adolescence, suggesting that “the seeds of wisdom lie in adolescence” [25] (p. 360). Adolescents are engaged in the challenges of becoming adults, which involve increasing responsibility for one’s own decisions and for interpreting and planning one’s life. Wisdom-related judgement and understanding are key to developing the skills needed for a successful transition to emerging adulthood [25]. Thus, there is a need to foster wisdom-related qualities that can be useful in daily life.

Staudinger and Baltes [26] argue that wisdom does not develop in a vacuum. They propose that social contexts in which wisdom is shared (such as mentorship) are important in the acquisition of wisdom. Thus, contact with individuals we consider wise can activate wisdom-related judgment and knowledge [26]. In recent years, there has been increasing attention to “wisdom education” for young people. Programs in this area have typically employed methods like discussion of well-known exemplars of wisdom (e.g., Gandhi), using fables and stories, and employing teaching techniques such as self-distancing [27].

However, little attention has been paid to a more obvious possibility: creating situations where young people actually listen to and process the wisdom and advice of older people. An underlying premise of the BCLT program is that interactions in which younger people receptively solicit older people’s wisdom, engage in structured discussions of what they have learned and present it to others, is a promising intervention strategy. Glück [28] proposes that it is important to extend the research on wisdom into real-world settings, noting that in many people’s minds, offering guidance and advice are the most typical manifestations of wisdom. In line with this thinking, the BCLT program focuses on wise behavior in real-life situations, and specifically in advice-giving interactions with older people.

Our conceptual framework centered on the role of intergenerational wisdom-sharing follows the theoretical model developed by Kessler and Staudinger [16]. Their model posits that intergenerational interaction fits with the developmental needs of both the young and the old, and thus can facilitate positive outcomes for both. Older adults are motived to engage in such interactions because of a developmental need to express generativity. Offering advice to a member of the younger generation can fulfill this function. Correspondingly, adolescents’ developmental tasks include forming an identity and developing a sense of purpose and meaning. They are motivated to search for knowledge about the self and life in general. As Kessler and Staudinger [16] note, youth’s identity formation and older adults’ generativity needs are complementary motivational concerns.

This theoretical framework suggests that advice-giving is a potential mechanism for effects of an intergenerational program. Further, advice-giving is likely to bolster the effects of intergenerational interaction, because it is perceived as a very meaningful transfer of experiential knowledge [16]. In the BCLT program, older people are placed in a position of expertise, allowing the youth participants to experience their attention and concerns. The model suggests that assigning expert status to older people will activate positive stereotypes of old age. In our program, the youth participants are trained in advance to appreciate the value of elder wisdom, thus increasing this effect.

Based on this framework, there is an important distinction between the BCLT program and numerous IGPs that involve interviewing older people about their life stories and reminiscences. The wisdom-sharing intervention employed in the present study differs from past research on intergenerational storytelling in several ways. First, storytelling interventions are often conducted within family contexts (cf. [29,30]) and do not always feature the young people’s involvement (cf. [31]). In contrast, the BCLT program connects youth with older adults from their local community.

Second, storytelling can involve anecdotes about minor experiences [32], whereas older adults’ life lessons and advice are more likely to draw on global, highly relevant experiences. Consequently, storytelling can lack mentoring benefits as life stories do not necessarily convey advice or wisdom in the way life lessons do. For instance, life history interventions [31] typically ask older adults to respond to questions such as “What is your earliest memory?” and “What clubs, groups, or organizations did you join?”. In contrast, we prompted wisdom-sharing with cues such as “What are some of the most important lessons you have learned over the course of your life that you would like to pass on to young people?”.

Third, storytelling interventions are less likely to involve an element of reciprocity. In storytelling programs, the focus is on offering older adults the opportunity to reminisce to an attentive listener. In contract, wisdom-sharing is meant to be of practical value to recipients. IGPs that include a give-and-take dynamic are more effective for participants [33]. Based on this conceptual model, the BCLT intervention incorporates active elements for youth in the form of designing questions to elicit specific advice from older people and developing a community presentation to share the relevant lessons learned.

### 1.3. Hypotheses

The present study focuses on outcomes for youth participants. The conceptual framework and design of the BCLT program are organized around testing benefits for youth participants, and program resources are largely devoted to training them. In part, this focus was determined by the funding source, a center for youth development that was primarily concerned with IGP outcomes for young people. In addition, the emphasis was determined to be a more critical need, given the plethora of evidence on the benefits of generativity for older people [2,8].

The evaluation focused on two types of outcomes for youth participants. First, we hypothesized positive changes in the treatment group (when compared to controls) for several proximal outcomes; that is, the more immediate results of program activities. One outcome assessed the degree to which youth participants felt confident in using the skills taught in the program, such as the ability to conduct an interview with an older person. The second proximal outcome was attitudes toward older people. In both cases, we hypothesized that the BCLT youth treatment group would improve significantly compared to the non-intervention youth control group. Third, a concrete outcome of attitude change toward older adults is whether a young person would find working with older people to be interesting or rewarding.

We also hypothesized positive treatment effects for distal outcomes; that is, anticipated final outcomes of the intervention that are further removed from the immediate effects. First, we hypothesized that experiencing and internalizing older people’s wisdom would significantly increase youth participants’ self-esteem as a result of BCLT participation. In addition, we hypothesized that interactions with older people around their lessons for living would enhance youth participants’ sense of purpose. Additional measures of health and well-being were not included in the study and should be employed in future research.

## 2. Materials and Methods

### 2.1. Design and Main Components of BCLT

A goal of the program development process was to incorporate evidence-based practices into the design of the BCLT program [8]. First, training was provided to the youth participants to prepare them for conducting interviews with the elders. The program leaders were trained by the university-based BCLT program staff in advance to conduct the youth training sessions. Orientation was also provided to the older participants to prepare them to share their life lessons. Second, the roles of young and older participants were clearly specified, including the importance of receiving and giving wisdom.

Third, we created a collaboration with a strong organizational partner that demonstrated enthusiastic buy-in to the program. We worked with the New York State 4-H Youth Development Program, which maintains a high-profile and trusted role in all counties in the state. Fourth, we identified a hypothesized mechanism for intervention effects (imparting advice for living from older to younger people). Fifth, we employed a rigorous research design to evaluate the BCLT program.

### 2.2. Program Components

BCLT is a manualized intervention that includes a set of standard components for all implementations (Table 1).

*Youth training*. BCLT begins with four hours of training prior to conducting face-to-face interviews with older adults. The goal of the training is to build skills and capacity for youth to interact with older adults. The training covers interview and communication skills tailored specifically to interactions with older adults, clarifying expectations when interacting with older adults, and methods to handle possible obstacles. An additional four hours of training were provided after the interviews to summarize the interview data and prepare a community report. The specific training curriculum is detailed in Table 2.

An important component of the training sessions is a guided opportunity for the youth participants to generate their own questions, based on their perceived need for life advice. Three questions are required to be included, because they were found to be particularly effective in eliciting life lessons from older people in prior research [7]. These include:

1. “What are some of the most important lessons you feel that you have learned over the course of your life? That is, if young people asked you, ‘What have you learned in your ____ years in this world’, what would you tell them?”
2. “Some people say they have had difficult or stressful experiences but that they have learned lessons from them. Is that true for you? Can you give examples of these experiences and what you learned?”
3. “What would you say are the major values or principles that you live by?”

A training session was devoted to generating additional questions derived from the youth participants experiences and interests. Questions emerged around typical challenges of adolescence, such as handling difficult interpersonal situations, romantic relationships, and current cultural and political issues. The following are examples of youth-generated questions:

- “Have you ever been bullied by someone? If so, what did you learn about responding to people that gave you a hard time?”
- “Were you ever discriminated against for something you believed in? What do you think is the best way to react to being in that situation?”
- “Do you have suggestions on ways to handle problems with siblings?”
- “What kind of advice do you have about being a good husband/wife or father/mother?”
- “What advice would you give youth about their role in politics today?”
- “Many young people today are involved in relationships that are more accepted today but still controversial, such as mixed-race or same-sex relationships. Is this something you can relate to and do you have advice for overcoming difficult relationship challenges?”
- “How did you know when you were in love? What are your recommendations on how to handle love?”

*Interviews with older adults.* Following the training, youth are paired with an older adult to conduct an hour-long individual interview. The older adults receive an orientation on the BCLT program prior to the interview. They are provided with background information on the program and they are given the list of questions they will be asked in the interview, allowing them time to reflect on the advice they may want to convey to a young person. Pilot-testing of the BCLT program demonstrated the need to provide older participants with this preparation so they could organize their thoughts prior to the interview.

*Summarizing individual and group findings*. Upon completion of the interview, the youth individually summarize the major lessons learned during their interviews and report their findings in a group meeting. With guidance from the group leader, participants analyze the qualitative responses of the older adults and come to consensus on the core set of lessons learned as a group. The lessons are synthesized into a single list of the most important advice received for each interview question. This component of BCLT treats the interviews as qualitative data and helps participants organize the lessons into general themes with characteristic examples.

*Community presentation*. The BLCT program culminates in an approximately one hour-long presentation for community members in which youth present the older participants’ lessons. The goal is to provide an opportunity for youth to publicly foster positive images of older people as sources of wisdom, thus reinforcing their individual experiences in the interviews. Guidance is provided for preparing the presentation and accompanying audiovisual materials. The older participants are invited to the community presentation to reinforce the importance of their participation and increasing their sense of generativity. Parents, friends, and members of community organizations are typically invited.

Thus, the BCLT intervention included several components that were likely to benefit middle school and high school youth, which are transferable to other situations. The training process provided them with useful interviewing and active listening skills. The organization of the questionnaire design and data analysis offered opportunities for cooperative learning. Further, the BCLT intervention helps youth to hone public speaking skills that are used in the community presentation. Measurement of the impact of these components is included in our measures of proximal outcomes.

### 2.3. Implementation Considerations

This article presents data using a randomized, controlled design that took place in five sites across New York State. However, prior to the implementation of this study, the BCLT program was implemented in six earlier sites and a detailed process evaluation was conducted at each site. The process evaluation included interviews with youth and older participants as well as program leaders to determine strengths of the BCLT program and potential improvements. Identical process evaluation activities took place in the randomized, controlled sites. From the accumulated intervention experience, several considerations emerged regarding program implementation.

### 2.4. Focus on Middle-School and High-School-Aged Youth

The program is designed for middle and high school age youth. This decision emerged from the experience of the initial implementations of the BCLT program, which included elementary school students. It was determined that the younger age group was challenged by the level of information in the training sessions and was much less able to conduct meaningful and detailed wisdom interviews. Adaptation for younger children is an important consideration for future program development.

### 2.5. Program Sponsorship

The BCLT program is designed to be led by a youth group, school, or similar organization in collaboration with a local aging service provider. Any interested organization that works with youth can initiate and implement the BCLT program. The BCLT leaders must have experience with teaching or program leadership with adolescents, as they facilitate the BCLT training. The benefit of using school classes or established youth programs (e.g., 4-H, Boys and Girls Clubs, after-school programs) is that recruitment can take place at one time and the BCLT program can be completed during regular program hours. In this case, BCLT becomes part of the curriculum or planned activities for that specific youth group. For example, the BCLT program can be implemented to complete a school service-learning requirement.

### 2.6. Ease of Implementation

Overall, reports from program leaders indicated that the BCLT intervention is an accessible program and is relatively easy to implement. Program leaders reported that the program manual is thorough and easy to read. Leaders also appreciated that the youth training offers a selection of exercises for any single training concept, which may include brainstorming, roleplaying, small-group discussions, watching videos, or other activities. In addition to endorsing the curriculum, program leaders universally reported that there is sufficient information about how to plan for and run the BCLT program, including detailed guidance regarding the logistical and administrative activities required to implement it. Further, program leaders appreciated that university-based BCLT program staff provided ongoing consultation and training to assist them in running the program.

Although the overall feedback from program leaders was overwhelmingly positive, there were also challenges that program leaders faced. Some leaders noted that finding and recruiting the youth and senior participants was time-consuming. We encouraged engaging community partners to assist with recruitment (e.g., schools, assisted living communities, senior centers), but some sites found such connections difficult to do based on their location.

Implementing the BCLT program requires that staff have sufficient time to manage program details successfully. Activities for program leaders include recruiting participants, obtaining parental consent for youth, and scheduling the training, interviews, and community presentation. These needs have led to the recommendation that the responsibility for leading the BCLT program be shared by two individuals, thus reducing the amount of administrative time required. Overall, challenges met by program leaders have been consistently mitigated by the thoroughness of the training manual and the consultative support from the university-based BCLT program staff team.

### 2.7. Program Cost

The cost of implementing the BCLT is low. The BCLT program manual is free and can be downloaded easily from the program website (http://citra-bclt.human.cornell.edu/). Some program leaders opted to provide refreshments for participants, a certificate of completion, and/or offered a small gift to participants, such as a gift card. These expenses amount to $200 or less. The most significant cost for running the BCLT program is the need to cover the program leader’s salary for the time they are implementing the BCLT. However, BCLT has been integrated into the regular programming activities of the program leaders such that their time is already accounted for. We estimate that implementing the BCLT requires up to 50 h of program leader time, with half of that time allocated to administrative tasks and half to the youth training, interviews, and the community presentation. Of course, two program leaders decrease the time required for each individual.

### 2.8. Fidelity 

We employed a number of mechanisms to endure treatment fidelity across sites, including the use of a standardized intervention manual; systematic training of program leaders in conducting the program as designed; regular meetings with the program leaders to review any protocol issues; and monitoring through direct observation of the community presentations.

### 2.9. Sample

BCLT program leaders were recruited from the New York State 4-H Youth Development Program. The National 4-H Program is the Youth Development Education Program of the Cooperative Extension Service, which in New York State is located at Cornell University. Cooperative Extension maintains offices in every county in New York State that sponsors 4-H. Trained 4-H Youth Development specialists oversee a range of hands-on educational activities, which are provided in local communities through local 4-H clubs, after-school programs, and summer camps. The NYS 4-H Program is an ideal partner, as it is a research-based organization that embraces the development and implementation of evidence-based approaches to youth programming.

Program leaders were selected in four New York State counties between 2016 and 2019, with one county conducting BCLT in two separate administrations in sequential years (five sites total). Given differences in programs and resources, considerable leeway was allowed in recruiting younger and older participants. In two sites, youth participants were recruited through the local 4-H program. In the remaining three sites, program leaders used existing connections with schools, including a high school leadership program, an after-school program, and student recruitment from social science classes. Older participants were recruited primarily through agency contacts of the program leaders, such as with local senior centers. Another source was older individuals involved with the county Cooperative Extension Association as volunteers.

A total of 93 youth participants were recruited, who were then randomized at the individual level into the BCLT intervention (*n* = 47) or into a non-intervention control group (*n* = 46). Control group participants did not receive the intervention and completed pre-test and post-test questionnaires. No statistically significant differences were found between the treatment and control groups on gender, age, race, or middle school/high school status. Sample characteristics for the treatment and control groups are presented in Table 3.

BCLT participants completed a pre-test questionnaire within two weeks of beginning the program and a post-test one week following the end of the program (approximately nine weeks from baseline). Shortly after completing the post-test, participants were contacted individually by telephone for a follow-up interview that solicited qualitative feedback about the program. Control participants took the pre- and post-test questionnaires at the same time as treatment participants; no follow-up interview was conducted for the control group. The pre-test included questions on participant demographics (age, gender, race, current grade in school). The treatment post-test included a feedback component that consisted of both quantitative and qualitative questions assessing program satisfaction.

### 2.10. Measures

*Perceived competency*. Youth responded to a 6-item Competency Scale that assessed their perceived ability to conduct and interpret interviews with older people. Using a 4-point scale anchored at (1) *Certain you can’t do it* and (4) *Certain you can do it*, youth rated how certain they were they could carry out activities such as listening attentively, taking notes, working in a team, and asking follow-up questions. Responses were summed up into a single score (6–24 points).

*Attitudes toward older people.* Perceptions concerning older adults were measured using the 9-item Attitudes Toward the Elderly Scale [34]. Youth indicated their agreement with statements such as “Most older people are in good health” on a 4-point scale anchored at (1) *Strongly disagree* and (4) *Agree*. Items were summed (9–36 points) so that higher scores indicate more positive views.

*Attitudes Toward Working with Older People Scale.* This scale measures the degree of agreement with statements regarding working with older people (e.g., “Working with older people is an interesting job”). The items are rated on a 4-point scale anchored at (1) *Strongly disagree* and (4) *Agree* [33].

*Purpose*. Purpose in life was assessed using the 6-item Life Engagement Test adapted from Scheier and colleagues [35]. Participants rated to what extent they agreed with statements such as “I value my activities a lot.” Responses were recorded on a 5-point Likert scale ranging from (1) *Strongly disagree* to (5) *Strongly agree* and summed (6–30 points) so that higher scores indicated stronger perceptions of purpose.

*Self-esteem.* On a 4-point scale ranging from (1) *Strongly disagree* to (4) *Strongly agree*, youth responded to five items from the Rosenberg Self-Esteem Scale [36], including “I take a positive attitude toward myself.” Items were summed (5–20), with higher scores reflecting higher levels of self-esteem.

### 2.11. Post-Test Only Measures

Youth responded to assessments of the program’s enjoyableness (1 = *Not at all enjoyable* to 4 = *Extremely enjoyable*), usefulness (1 = *Not at all enjoyable* to 4 = *Extremely enjoyable*), and perceived benefit for the older participants (1 = *Not at all* to 4 = *A lot*). They were also asked whether they would recommend the program to other people like them (1 = *No*, 2 = *Yes*). Open-ended questions included “What did you like most about [interviewing an older person/being interviewed by a young person]?” and “Please list some things you got out of participating in the Building a Community Legacy Together (BCLT) program.”

### 2.12. Statistical Models and Methods

We obtained descriptive statistics for the sociodemographic variables and outcome variables at baseline. The core model for each outcome for evaluation of the intervention included treatment (2 levels—control and BCLT intervention) and time of assessment (2 levels—pre-tests at baseline and post-tests one week later at the completion of the intervention) as fixed classification factors, the interaction of these 2 factors, and individuals as levels of a random classification factor. The models also included *a priori* additional sociodemographic independent variables, specifically gender and current grade in school (a collapsing of grade levels to 2 categories, middle school grades 6–8 and high school grades 9–12, as fixed classification factors for the youth outcomes).

The interaction of each of the additional variables—as well as other variables such as site—with treatment and time was also examined. This involved a 3-way interaction of fixed factors for the additional categorical variables and homogeneity of regressions of the covariate by levels of treatment and time for the additional quantitative variables [37]. There was no coherent significance for interactions, and the final models presented do not include interactions.

Analysis was by general linear mixed models assuming normality with unstructured error. Degrees of freedom were computed using the first-order Kenward-Rogers method [38]. The key test of the effectiveness of the intervention is the treatment × time interaction. Table 4 shows LS means and standard errors and differences of means and Ps for tests of those differences. Missing data at the post-test were handled using the maximum likelihood estimation of the mixed models.

## 3. Results

### 3.1. Outcome Analysis

The primary analyses for evaluation of the program are shown in Table 4. The tables show, for each outcome, the treatment × time (2 × 2) adjusted means and SEs, mean differences by time and by treatment (the marginals), and in the lower-right cell the P for the treatment × time interaction.

As can be seen from Table 4, the test of treatment effect for four of the five youth outcomes are significant and in the direction of positive program effects—perceived competency in interaction with older people (*p* = 0.0002), attitudes toward older people (*p* ≤ 0.0001), interest in working with older people (*p* = 0.007), and purpose in life (*p* = 0.053). Self-esteem, although in a direction that favors the program, did not reach statistical significance (*p* = 0.196). Interactions among treatment, time, and sociodemographic variables were examined fully. There was no coherent pattern, and interactions are not included in the final models. There were no differences by site.

### 3.2. Subjective Evaluation of Program

Subjective evaluations of program experience were extremely positive based on the post-intervention questionnaire. In their overall evaluation of the program, 97 percent of youth participants reported that the BCLT program was enjoyable. All youth participants felt the experience was useful, and 97 percent also believed the program was useful to the older participants. Almost all (91 percent) of youth participants would recommend the program to someone else.

Responses to open-ended questions found similarly positive responses, with most youth participants specifically referring to the benefits of receiving life advice. Examples include:

- “Having patience to talk with me, and help me with some advice for the future.”
- “Hearing about their past and the things they took away from their experience.”
- “I liked gaining advice from someone who has actually lived through life and gaining advice on my career.”
- “Hearing their ideas about how to deal with challenges in life.”

These high levels of participant satisfaction provide further evidence of successful implementation of the program components.

## 4. Discussion

The findings from this evaluation provide encouraging evidence for the benefits of wisdom sharing as a mechanism for positive effects in an IGP. The pattern for all outcomes was an increase in means over time for both control and treatment groups, but a considerably larger increase for program participants. The randomized, controlled design lends confidence that these effects emerged from program participation. In addition, the subjective evaluations of the value of the program were very high. Although further replication is needed, these quantitative findings—supported by the overwhelmingly positive program satisfaction data—provide support for the effectiveness of the BCLT program.

The program’s focus on obtaining elder wisdom can address a potential problem in intergenerational programs: the issue of “generative mismatch.” Because of rapid social and technological change, the skills and knowledge of older adults could be seen by young people as being outdated or irrelevant [39,40]. However, life advice from older adults that is targeted to identified needs of younger people may circumvent this challenge. In the BCLT intervention, questions are designed by the youth participants that enhance the relevance of the advice they receive, thus reducing the experiential mismatch. The qualitative data we presented suggests that the older participants’ wisdom connected well with the experiences of the youth participants.

There are a number of limitations to the study, however, that point to needed directions for future research. First, we were able to assess only a limited range of outcomes, and over a relatively short time period. A major priority for future research should be illuminating the effects of IGP participation on physical and mental health outcomes. We were able to assess two domains that are associated with greater well-being. Sense of purpose improved significantly, and self-esteem also improved but failed to reach statistical significance. Multiple post-tests that focus on health-related outcomes are needed, especially given research findings discussed earlier suggesting that wisdom interventions may improve health.

Second, this study compared youth participants in the BCLT intervention with a non-intervention control group. The resources for this project did not allow us to include an additional arm of the study that would more firmly establish wisdom-sharing as the mechanism of effects. To identify the relative contribution of wisdom-sharing in relation to the program’s other facets (e.g., contact with older people), future studies could include a comparison arm focused on contact and activities other than wisdom-sharing.

Third, it is likely that the sample of youth participants disproportionately included individuals who already had some interest in older people and were receptive to their life advice. It is very difficult to avoid such potential bias in a study that involves recruitment in real-world settings. The randomization of youth participants into treatment and control groups ensures that this bias did not influence the intervention results.

Fourth, this study is limited by its focus on the youth participants. Older participants were engaged as advice-givers and received an orientation specific to that activity. One reason for this design decision was to allow wisdom-sharing to take place more naturally, without extensive coaching and preparation beforehand of older participants. Further, as noted earlier, a priority was testing the effects of the receipt of advice for youth, given that it is an understudied phenomenon. Subjective assessments of the program were very positive on the part of the older adults, who found BCLT participation to be rewarding and enjoyable. Program modifications are now underway that expand the preparation, training, and engagement of older participants with the goal of increasing positive outcomes for them (and possibly, as a result, for the youth participants).

## 5. Conclusions

Interest in IGPs has proliferated over the past decade, making the field an exciting one for both researchers and practitioners. As studies embrace more sophisticated research designs and theory-based interventions, guidance for designing the most effective programs will be enhanced. The present study incorporated a number of recommendations for rigorous evaluation of IGPs, including specifying the mechanism for effects, ensuring treatment fidelity through a manualized intervention, and using a randomized, controlled design. The findings of this study indicate that the BCLT had positive effects for youth participants. Thus, the research supports the development and promotion of programs that focus on the transfer of wisdom from older to younger persons.

The study also indicates the potential for linking the growing field of wisdom research to that on IGPs. To date, there has been limited interaction between these two perspectives, with few wisdom researchers conducting studies that include real-world intergenerational interactions. The results of our research point to the potential of research translation between the two fields, which can not only lead to the development of new, evidence-based IGPs, but also can contribute to basic knowledge of how wisdom is transmitted and its potential effects on health and well-being.

## Figures and Tables

**Table 1:** Components of the BCLT Program.

Overview	
The BCLT program trains youth to interview older adults (ages 65 and over) about their life wisdom, including overcoming adversity, dealing with crises, and facing life’s uncertainties, as well as specific questions of particular interest to the youth. The program exposes the youth to social science methods such as interviewing skills and techniques to interpret, analyze, and present interview data.	
**Component**	**Description**
a. Youth training	Training is provided in preparation for the interviews with older adults.
b. Interviews with older adults	Each youth participant conducts a life wisdom interview with an older adult lasting approximately 1 h and recorded.
c. Summarizing individual and group findings	Youth participants create individual interview summaries that include identifying the lessons offered. The group then compiles the interview data and prioritizes the lessons.
d. Community presentation	Youth participants create and lead a public presentation of the findings from their interviews, focusing on the life lessons of older adults in their community.

**Table 2:** BCLT youth training curriculum.

A. What Is the BCLT Program All about? (2 h)	
Introductions	A roleplay exercise where youth pair up and interview each other using predetermined questions. The goal of this exercise is to give youth participants the chance to get to know each other and get a sense of what it is like to interview and be interviewed.
2. Overview of the BCLT program	This unit provides youth participants with background information on the program and helps them understand the difference between oral history and life wisdom interviews.
3. Ageism and elder wisdom	Through brainstorming, follow-up discussion, and some short lecture, youth participants collectively define ageism, identify stereotypes of older adults, and further explore the concept of elder wisdom.
4. Developing the interview questions	Youth participants review background information on qualitative interviewing, learn the difference between closed-ended and open-ended questions, become familiar with possible interview questions and answers, and finalize their own lists of questions.
**B. Interviewing skills (2 h)**	
Introduce yourself	This is a quick exercise designed to give youth participants a chance to practice how to introduce themselves to their interviewees. Introductions include the youth’s name, grade, school, and one sentence about why they are participating in the BCLT.
2. Basic listening skills	Youth participants learn what active listening involves and why listening in an interview is important to build rapport with the interviewee.
3. Specific interviewing techniques	In preparation for the interviews with the older adults, the youth learn to identify and then practice specific interviewing skills.
4. Follow-up and probing questions	Youth participants learn the interview technique of asking follow-up questions and probing for more information.
5. Roleplay the interview	This session includes opportunities for the youth to roleplay the interviews using all the skills and techniques they just learned in the previous exercises. Through roleplaying, the youth become familiar with the questions, learn to use follow-up questions consistently, and overcome nervousness.
**C. Unpacking the data (2 h)**	
Summarize group findings	Prior to this training session, youth participants review the interview recording and list the life lessons offered by their interviewee. As a group, the data are then summarized by listing the lessons, counting the frequency that any one lesson is offered and determining the importance of each lesson to the youth.
**D. Preparing a community presentation (2 h)**	
Create a presentation to share with the community	The final training session is devoted to planning a community presentation.

**Table 3:** Selected characteristics of the youth participants (*N* = 93).

Variable	Treatment (*n* = 47)		Control (*n* = 46)		Statistic
	*n*	%	*n*	%	
Gender					
Female	21	46.7	16	34	
Male	24	53.3	31	66.6	*p* = 0.15
Grade in school					
Middle school	14	31.8	14	29.8	
High school	30	68.2	33	70.2	*p* = 0.51
Race					
White	28	60.9	34	72.3	
Black	4	8.6	3	6.4	
Asian	8	17.4	8	17	
Other	6	13	3	4.3	*p* = 0.44
Age					
13–14	7	15.9	7	14.9	
15	6	13.6	6	12.8	
16	9	20.5	6	12.8	
17–18	22	50	28	59.6	*p* = 0.75

**Table 4:** Evaluation of effects of the intervention for youth participants.

Change Over Time			
Variable	Pre-Test	Post-Test	Post-Test–Pre-Test Mean (*p*-Value)
Perceived competency			
Control	20.36 (0.336)	20.48 (0.344)	0.11 (0.722)
Treatment	20.14 (0.333)	21.98 (0.336)	1.84 (<0.0001)
T–C difference (*p*-value)	−0.22 (0.613)	1.50 (0.001)	−1.72 (0.0002)
Attitudes toward older people			
Control	25.34 (0.522)	26.44 (0.538)	1.10 (0.061)
Treatement	23.87 (0.518)	28.70 (0.523)	4.84 (<0.0001)
T–C difference (*p*-value)	1.47 (0.040)	2.27 (0.002)	−3.73 (<0.0001)
Interest in working with older people			
Control	22.38 (0.436)	22.65 (0.446)	0.27 (0.403)
Treatement	22.33 (0.432)	24.19 (0.435)	1.86 (0.405)
T–C difference (*p*-value)	−0.05 (0.927)	1.54 (0.009)	−1.59 (0.007)
Purpose			
Control	24.19 (0.554)	24.23 (0.560)	0.04 (0.927)
Treatement	24.77 (0.546)	25.97 (0.551)	1.21 (0.005)
T–C difference (*p*-value)	0.58 (0.406)	1.75 (0.014)	−1.17 (0.053)
Self-esteem			
Control	16.51 (0.354)	17.21 (0.362)	0.70 (0.034)
Treatement	16.35 (0.351)	17.65 (0.356)	1.30 (0.0002)
T–C difference (*p*-value)	−0.16 (0.725)	0.44 (0.355)	−0.60 (0.196)

Note: Table entries are means (SEs) in the 2 × 2 (treatment × time) upper left quadrant for each dependent variable and mean differences (Ps) in the other three quadrants. The entries in the bottom right cell for each dependent variable represent the tests of treatment effects. The model for each outcome also includes gender and grade level (middle school-aged versus high school-aged students) as additional fixed classification factors, and individuals as levels of a random classification factor.

## Data Availability

Requests for use of the data should be directed to the first author.

## References

[1] U.S. Census Bureau (2018). The population 65 years and older in the United States. ACS Am. Community Surv. Rep..

[2] Whitehouse P.J. (2017). Learning among generations—from intergenerational to intergenerative. Generations.

[3] Drury L., Hutchison P., Abrams D. (2016). Direct and extended intergenerational contact and young people’s attitudes towards older adults. Br. J. Soc. Psychol..

[4] Bruya B., Ardelt M. (2018). Wisdom can be taught: A proof-of-concept study for fostering wisdom in the classroom. Learn. Instr..

[5] Sharma A., Dewangan R.L. (2017). Can wisdom be fostered: Time to test the model of wisdom. Cogent Psychol..

[6] Sternberg R.J., Sternberg R.J., Nusbaum H.C., Glück J. (2019). Where have all the flowers of wisdom gone? An analysis of teaching for wisdom over the years. Applying Wisdom to Contemporary World Problems.

[7] Pillemer K. (2011). 30 Lessons for Living: Tried and True Advice from the Wisest Americans.

[8] Jarrott S.E., Scrivano R.M., Park C., Mendoza A.N. (2021). Implementation of evidence-based practices in intergenerational programming: A scoping review. Res. Aging.

[9] DeMichelis C., Ferrari M., Rozin T., Stern B. (2015). Teaching for wisdom in an intergenerational high-school-English class. Educ. Gerontol..

[10] Peters R., Ee N., Ward S.A., Kenning G., Radford K., Goldwater M., Dodge H., Lewis E., Xu Y., Kudrna G. (2021). Intergenerational programmes bringing together community dwelling non-familial older adults and children: A systematic review. Arch. Gerontol. Geriatr..

[11] Giraudeau C., Bailly N. (2019). Intergenerational programs: What can school-age children and older people expect from them? A systematic review. Eur. J. Ageing.

[12] Chua P.H., Jung Y., Lwin M.O., Theng Y.L. (2013). Let’s play together: Effects of video-game play on intergenerational perceptions among youth and elderly participants. Comput. Hum. Behav..

[13] Sun Q., Lou V.W., Dai A., To C., Wong S.Y. (2018). The effectiveness of the Young–Old Link and Growth intergenerational program in reducing age stereotypes. Res. Soc. Work Pract..

[14] Burnes D., Sheppard C., Henderson C.R., Wassel M., Cope R., Barber C., Pillemer K. (2019). Interventions to reduce ageism against older adults: A systematic review and meta-analysis. Am. J. Public Health.

[15] Chorn Dunham C., Casadonte D. (2009). Children’s attitudes and classroom interaction in an intergenerational education program. Educ. Gerontol..

[16] Kessler E.M., Staudinger U.M. (2007). Intergenerational potential: Effects of social interaction between older adults and adolescents. Psychol. Aging.

[17] Kassab C., Vance L. (1999). An assessment of the effectiveness of an intergenerational program for youth. Psychol. Rep..

[18] Angus R., Hughes T. (2017). School climate, connectedness and academic achievement: Examining positive impacts from high school mentoring services. ELRDR.

[19] Taylor A.S., Losciuto L., Fox M., Hilbert S.M., Sonkowsky M. (1999). The mentoring factor: Evaluation of the across ages’ intergenerational approach to drug abuse prevention. Child Youth Serv..

[20] Park A.L. (2015). The effects of intergenerational programmes on children and young people. Int. J. Sch. Cogn. Psychol..

[21] De Souza E.M., Grundy E. (2007). Intergenerational interaction, social capital and health: Results from a randomised controlled trial in Brazil. Soc. Sci. Med..

[22] Kim J., Lee J. (2018). Intergenerational program for nursing home residents and adolescents in Korea. J. Gerontol. Nurs..

[23] Carstensen L.L., Freedman M., Larson C. (2016). Hidden in Plain Sight: How Intergenerational Relationships Can Transform Our Future.

[24] Tabuchi M., Nakagawa T., Miura A., Gondo Y. (2015). Generativity and interaction between the old and young: The role of perceived respect and perceived rejection. Gerontologist.

[25] Pasupathi M., Staudinger U.M., Baltes P.B. (2001). Seeds of wisdom: Adolescents’ knowledge and judgment about difficult life problems. Dev. Psychol..

[26] Staudinger U.M., Baltes P.B. (1996). Interactive minds: A facilitative setting for wisdom-related performance?. J. Pers. Soc. Psychol..

[27] Huynh A.C., Grossmann I. (2020). A pathway for wisdom-focused education. J. Moral Edu..

[28] Glück J. (2020). The important difference between psychologists’ labs and real life: Evaluating the validity of models of wisdom. Psychol. Inq..

[29] Jones J., Ackerman M.S. (2018). Co-constructing family memory: Understanding the intergenerational practices of passing on family stories. Proceedings of the 2018 CHI Conference on Human Factors in Computing System.

[30] Wallbaum T., Matviienko A., Ananthanarayan S., Olsson T., Heuten W., Boll S.C.J. (2018). Supporting communication between grandparents and grandchildren through tangible storytelling systems. Proceedings of the 2018 CHI Conference on Human Factors in Computing System.

[31] Li C., Hu J., Hengeveld B., Hummels C. (2020). Facilitating intergenerational storytelling for older adults in the nursing home: A case study. J. Ambient Intell. Smart Environ..

[32] Kennedy G.E. (1992). Shared activities of grandparents and grandchildren. Psychol. Rep..

[33] Hernandez C.R., Gonzalez M.Z. (2008). Effects of intergenerational interaction on aging. Educ. Gerontol..

[34] Pillemer K., Schultz L., Bleiberg Seperson S., Hegeman C. (2002). Evaluation of the Student Assisted Independent Living (SAIL) service learning project. Elder Care and Service Learning: A Handbook.

[35] Scheier M.F., Wrosch C., Baum A., Cohen S., Martire L.M., Matthews K.A., Schulz R., Zdaniuk B. (2006). The life engagement test: Assessing purpose in life. J. Behav. Med..

[36] Rosenberg M. (1965). Society and the Adolescent Self-Image.

[37] Henderson C. (1982). Analysis of covariance in the mixed model: Higher level, nonhomogeneous, and random regressions. Biometrics.

[38] Kenward M.G., Roger J.H. (1997). Small sample inference for fixed effects from restricted maximum likelihood. Biometrics.

[39] McAdams D.P., Hart H.M., Maruna S., McAdams D.P., de St. Aubin W. (1998). The anatomy of generativity. Generativity and Adult Development: How and Why We Care for the Next Generation.

[40] Cheng S.T. (2009). Generativity in later life: Perceived respect from younger generations as a determinant of goal disengagement and psychological well-being. J. Gerontol. Ser. B.

